# Syntrophic acetate oxidation replaces acetoclastic methanogenesis during thermophilic digestion of biowaste

**DOI:** 10.1186/s40168-020-00862-5

**Published:** 2020-07-03

**Authors:** Stefan Dyksma, Lukas Jansen, Claudia Gallert

**Affiliations:** Faculty of Technology, Microbiology – Biotechnology, University of Applied Sciences Emden/Leer, Emden, Germany

**Keywords:** Anaerobic digestion, Metagenome, 16S rRNA gene amplicons, Enrichment cultures, Syntrophic acetate-oxidizing bacteria

## Abstract

**Background:**

Anaerobic digestion (AD) is a globally important technology for effective waste and wastewater management. In AD, microorganisms interact in a complex food web for the production of biogas. Here, acetoclastic methanogens and syntrophic acetate-oxidizing bacteria (SAOB) compete for acetate, a major intermediate in the mineralization of organic matter. Although evidence is emerging that syntrophic acetate oxidation is an important pathway for methane production, knowledge about the SAOB is still very limited.

**Results:**

A metabolic reconstruction of metagenome-assembled genomes (MAGs) from a thermophilic solid state biowaste digester covered the basic functions of the biogas microbial community. *Firmicutes* was the most abundant phylum in the metagenome (53%) harboring species that take place in various functions ranging from the hydrolysis of polymers to syntrophic acetate oxidation. The Wood-Ljungdahl pathway for syntrophic acetate oxidation and corresponding genes for energy conservation were identified in a *Dethiobacteraceae* MAG that is phylogenetically related to known SAOB. 16S rRNA gene amplicon sequencing and enrichment cultivation consistently identified the uncultured *Dethiobacteraceae* together with *Syntrophaceticus*, *Tepidanaerobacter*, and unclassified *Clostridia* as members of a potential acetate-oxidizing core community in nine full-scare digesters, whereas acetoclastic methanogens were barely detected.

**Conclusions:**

Results presented here provide new insights into a remarkable anaerobic digestion ecosystem where acetate catabolism is mainly realized by *Bacteria*. Metagenomics and enrichment cultivation revealed a core community of diverse and novel uncultured acetate-oxidizing bacteria and point to a particular niche for them in dry fermentation of biowaste. Their genomic repertoire suggests metabolic plasticity besides the potential for syntrophic acetate oxidation.

Video Abstract

## Background

Acetate is a central intermediate in the mineralization of organic matter. It is a quantitatively important product that is released by fermentative microorganisms, and its removal is vital for carbon cycling. In methanogenic environments, the fate of acetate is either to be consumed by acetoclastic methanogens that directly use it for methanogenesis (reaction 1) or it is oxidized by syntrophic acetate-oxidizing bacteria (SAOB). Syntrophic acetate oxidation is dependent on interspecies hydrogen and/or formate transfer in which the syntrophic partner consumes the fermentation products, e.g., a hydrogenotrophic methanogen (reaction 2 and 3). The overall reaction for syntrophic acetate oxidation has the same stoichiometry as acetoclastic methanogenesis; however, the small amount of energy that is produced by this process (∆G^0’^ = − 31.0 kJ/mol) [1] needs to be shared by two microorganisms that have to struggle close to the thermodynamic equilibrium.

1. CH_3_COO^−^ + H_2_O → CH_4_ + HCO_3_^−^ (∆G^0’^ = − 31.0 kJ/mol)
2. CH_3_COO^−^ + 4H_2_O → 2HCO_3_^−^ + 4H_2_ + H^+^ (∆G^0’^ = + 104.6 kJ/mol)
3. 4H_2_ + HCO_3_^−^ + H^+^ → CH_4_ + 3H_2_O (∆G^0’^ = -135.6 kJ/mol)

Anaerobic digestion (AD) is a beneficial technology to treat wastewater sludge and biowaste for the production of renewable energy in the form of methane. Here, acetate is an important precursor for methanogenesis [2]. The constraint that SAOB need to share the energy with a partner methanogen suggests a disadvantage over acetoclastic methanogens such as *Methanosarcina* and *Methanosaeta* [3]. Hydrogenotrophic and acetoclastic methanogens generally coexist in AD communities [4–6] while the *Methanosarcina* were regularly identified as most abundant and omnipresent [7]. Unlike *Methanosaeta* that is strictly dependent on acetate, the methane metabolism of *Methanosarcina* is much more versatile including substrates such as methanol and methylamines as well as hydrogenotrophic growth [8]. Several factors determine the ecological niche of acetoclastic methanogens and their competitors, the SAOB. At elevated temperature, syntrophic acetate oxidation becomes thermodynamically more favorable [9]. The competitive success of SAOB in AD is further affected by the dilution rate and fatty acid concentrations [10–13]. Moreover, high ammonia levels that can be released during the degradation of proteinaceous material were reported to affect or inhibit methanogenesis [14–17]. Unionized free ammonia (NH_3_) is toxic to microorganisms [18]; however, acetoclastic methanogens are generally more sensitive to ammonia than the hydrogenotrophic ones [19–21]. Under these high ammonia conditions, syntrophic acetate oxidation can become the dominant process for acetate consumption although acetoclastic methanogenesis proceed concurrently [22–27].

Acetate-consuming methanogens are fairly well studied, but comparatively, little is known about the physiology and ecology of SAOB. Only few isolated species are described so far: the mesophilic *Clostridium ultunense* [28], *Syntrophaceticus schinkii* [29], *Ca*. Syntrophonatronum acetioxidans [30], the thermotolerant *Tepidanaerobacter acetatoxydans* [31], and the thermophilic *Pseudothermotoga lettingae* [32] and *Thermacetogenium phaeum* [33]. All described species can also grow axenically as heterotrophs-producing acetate from a variety of substrates or even grow autotrophically through homoacetogenesis as reported for *T. phaeum* [33]. Although the majority of SAOB were isolated from biogas reactors, syntrophic acetate oxidation is important for carbon cycling in natural environments beyond AD such as sediments [34–36], oil-reservoirs [37], and soils [38, 39].

In this study, we targeted microorganisms involved in acetate turnover from nine thermophilic biogas reactors that treat biowaste in dry-fermentation. 16S rRNA gene amplicon sequencing consistently identified *Syntrophaceticus* while acetoclasic methanogens were barely detected. In enrichment cultures, known genera of SAOB (*Tepidanaerobacter* and *Syntrophaceticus*) were detected but also *Clostridia* distantly related to *Caldicoprobacter* were highly enriched. Potential mechanisms of carbon cycling in these biogas reactors were further characterized using metagenome sequencing. Therefore, we built an automated metagenome analysis pipeline embedded in a Snakemake workflow. Along with the qualities of Snakemake [40], this pipeline is transferable, easily customizable, and scalable for much more complex datasets. Raw read processing, assembly, binning, annotation all the way to phylogeny of metagenome-assembled genomes (MAGs), and detailed reporting are automated in a customizable workflow. It is available at github.com/KnuttPipeline. Together, the enrichment cultures and the cultivation-independent approaches gained insights into an unusual methanogenic community of AD systems that represent a particular niche for syntrophic acetate-oxidizing bacteria.

## Results and discussion

### Genomic potential in thermophilic dry fermentation of biowaste

**Fig. 1:**
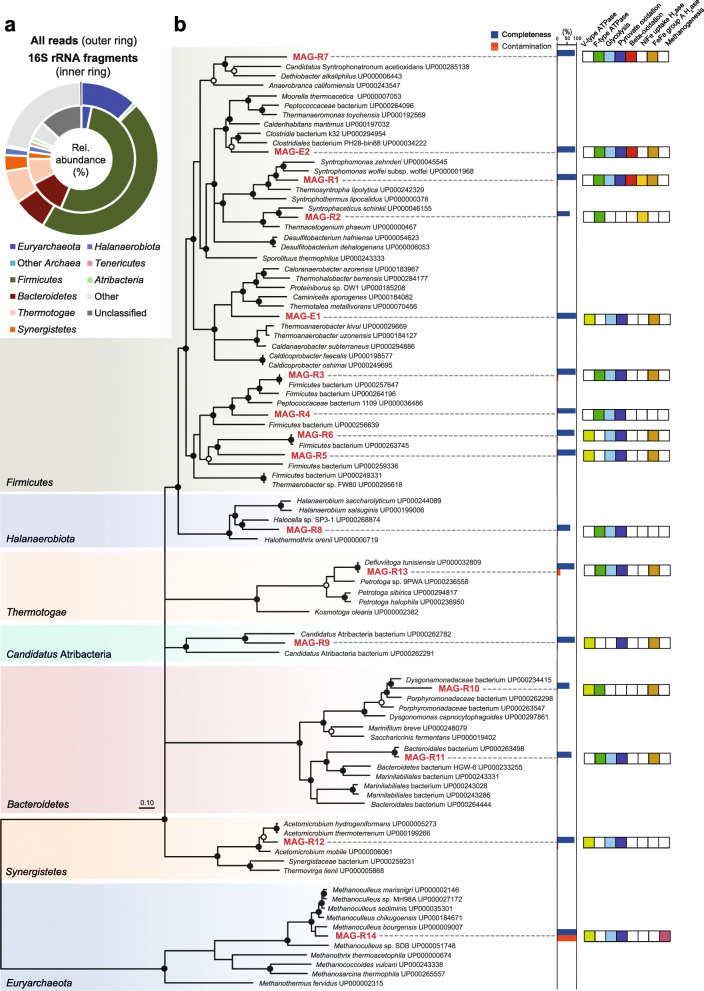
Taxonomic assignment of quality trimmed metagenome reads summarized on phylum level (**a**) based on total reads (outer ring) and 16S rRNA gene fragments (inner ring). Phylogeny of metagenome assembled genomes based on concatenated marker protein sequences and selected metabolic functions encoded in their genomes (**b**). Further information about contamination and completeness of the MAGs is provided in Additional file 1: Table S1. RAxML bootstrap support > 70% is indicated by open circles, and bootstrap support > 90% is indicated by closed circles. Multifurcations were introduced for branches with bootstrap support < 50%

A total of ~ 645 megabases of quality trimmed metagenomic sequencing data was recovered from a thermophilic digester that treats biowaste. Both 16S rRNA gene fragments extracted from the metagenome and total quality trimmed reads were used for taxonomic assignment (Fig. 1a, Additional file 2: Supplementary Methods). Classification of reads that could be mapped to a 16S rRNA gene reference database revealed *Firmicutes* as dominant bacterial phylum (53.2%) followed by *Bacteroidetes* (11.1%) and *Thermotogae* (7.7%). All other bacterial phyla together accounted for 11.8% (Fig. 1a). *Archaea* contributed 3.1% to the prokaryotic community and was strikingly dominated by the genera *Methanoculleus* (2.6%) and *Methanothermobacter* (0.4%) (Fig. 1a, Additional file 1: Fig. S2). Assembly and binning allowed the reconstruction of 14 MAGs that showed more than 60% of marker gene completeness although the sequencing depth was rather low, which is indicated by the average assembly coverage of 4.7-fold. MAGs could be recovered from almost all major phyla that were identified from total metagenome reads (Fig. 1a), and 57% of the metagenome reads could be mapped back to the MAGs. The phyla *Atribacteria*, *Bacteroidetes*, *Halanaerobiota*, *Firmicutes*, *Synergistetes*, and *Thermotogae* were represented in the metagenome by at least one medium-quality MAG (> 50% complete). The majority of MAGs (7 out of 14) affiliated with the dominant phylum *Firmicutes*. Most of the MAGs contained less than 4% contamination, though the *Methanoculleus* MAG-R14 showed a duplication of almost all single copy marker genes (Additional file 1: Table S1). Moreover, the *Defluviitoga* MAG-R13 (*Thermotogae*) contained 14% contamination from closely related species. Concatenated ribosomal proteins and the taxonomic assignment of the annotated coding sequences (CDS) were used for a more detailed phylogenetic identification of the MAGs (Fig. 1b). A comparison of the common modules and those that were mostly absent suggests that fermentation rather than respiration account for the majority of carbon mineralization (Fig. 1b), which agrees with the very low concentration of common electron acceptors typically found in AD systems. Moreover, the most widespread hydrogenases found in the MAGs and most abundant in unbinned metagenome sequences were electron confurcating group A3 [FeFe] hydrogenases (Additional file 1: Fig. S1) that couple the fermentative evolution of hydrogen from NADH with the oxidation of reduced ferredoxin [41].

**Fig. 2:**
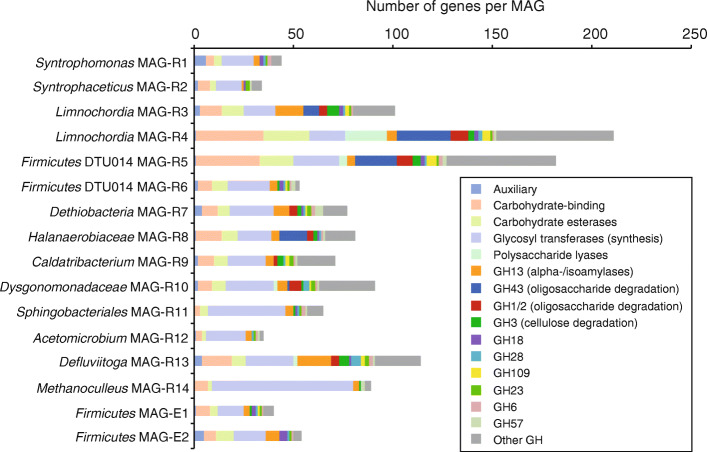
Number and groups of carbohydrate active enzymes identified in the MAGs. Major functions associated with selected groups are indicated. Glycosyl hydrolase families (GH) primarily include carbohydrate degrading enzymes

AD of organic matter starts with the depolymerization of macromolecular material. Biowaste can be rich in polymers derived from plant material like cellulose. The large fraction of *Defluviitoga* (MAG-R13) in this system indicates the relevance of these bacteria for the hydrolysis of polysaccharides. *Defluviitoga* utilize a remarkable variety of complex carbohydrates such as xylan, pullulan, lichenan, galactan, chitin and cellulose, among others [42, 43] producing acetate, ethanol, hydrogen, and carbon dioxide. *Defluviitoga tunisiensis* was also identified as widespread and abundant suggesting a central role in thermophilic biogas reactors [44]. Among the highest number of intracellular and extracellular carbohydrate-active enzymes (CAZymes) were identified in the *Defluviitoga* MAG-R13, which further support their importance for the initial breakdown of macromolecular compounds (Fig. 2). In addition, genes involved in oligosaccharide and sugar degradation as well as the relevant transporters were prevalent among the bacterial MAGs. This is in coherence with a high functional redundancy that has been reported for the first steps of AD and a gradually more specialized community in the later steps of AD [44–46]. Several complete modules for amino acid metabolism and degradation were detected in the *Sphingobacteriales* MAG-R11 suggesting uncultured *Bacteroidetes* as degrader of proteinaceous material besides their capability to utilize carbohydrates (Fig. 2). Genes for dissimilatory sulfur metabolism (sat and apr) were only identified in *Halanaerobiaceae* MAG-R8, but their sulfur-metabolizing capacity remains unclear as closely relates species such as *Halocella cellulolytica* and *Halothermothrix orenii* were described as obligate-fermenting bacteria [47, 48]. The final step of AD in this system is facilitated by hydrogenotrophic methanogens of the genera *Methanoculleus* (MAG-R14) and *Methanothermobacter* (Fig. 1a, Additional file 1: Fig. S2) that potentially act as syntrophic partner for the bacteria described below.

### Metagenomic perspective on syntrophy and microbial interactions

**Fig. 3:**
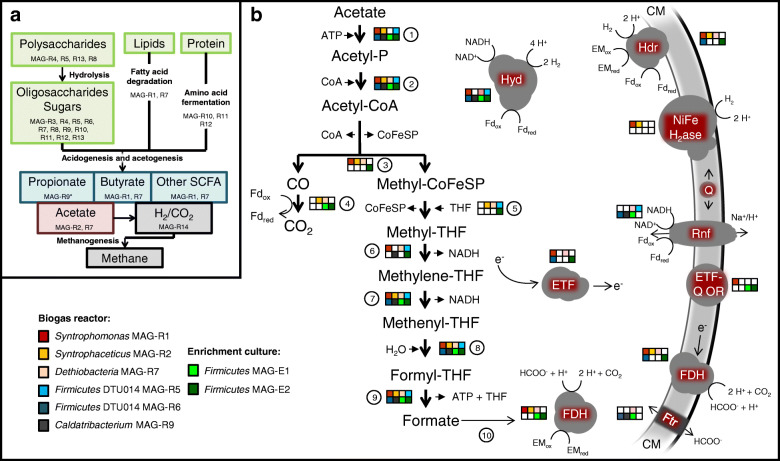
Putative functions of the MAGs in the anaerobic digestion food web (**a**). The asterisk (MAG-R9) indicates a potential function that merely proposed in literature (see text for details). Wood-Ljungdahl pathway genes in MAGs of syntrophic and putatively syntrophic bacteria as well as the corresponding energy-conserving mechanisms (**b**). Hyd, [FeFe] hydrogenase group A3; Hdr, heterodisulfide reductase; NiFe H2ase, energy-conserving group 1a [NiFe] hydrogenase; Rnf, Na^+^/H^+^ translocating ferredoxin:NAD^+^ oxidoreductase; Q, quinone; ETF, electron transfer flavoprotein; ETF-Q OR, electron transfer flavoprotein/quinone oxidoreductase; FDH, formate dehydrogenase; Ftr, formate transporter; EM, electron mediator; CM, cytoplasmic membrane; THF, tetrahydrofolate. Genes involved in the Wood-Ljungdahl pathway are acetate kinase (1), phosphotransacetylase (2), acetyl-CoA synthase complex including corrinoid protein (CoFeSP) (3), carbon monoxide dehydrogenase (4), methyltransferase (5), methylene-THF reductase (6), methylene-THF dehydrogenase (7), methenyl-THF cyclohydrolase (8), formyl-THF synthethase (9), and formate dehydrogenase (10)

Several MAGs were assigned to syntrophic or putatively syntrophic bacteria (Fig. 3). Here, we particularly focused on SAOB as acetoclastic methanogens were not detected in the MAGs and unassembled metagenome reads (Fig. 1), which suggests that acetate is mainly turned over by bacteria.

Evidence from biochemistry and transcriptomics revealed that some SAOB use the reversed Wood-Ljungdahl (WL) pathway for acetate oxidation [49–52]. The WL pathway can operate in both the reductive and oxidative direction, and diverse acetogenic bacteria use this pathway to produce acetyl-CoA from CO_2_ with various electron donors or use the pathway as electron sink during carbohydrate or organic acid fermentation [53]. WL pathway genes of SAOB were usually scattered in the genome not organized in an operon structure [54], and based on gene homology, it is further not possible to predict if the pathway could be reversed for acetate oxidation [52]. Therefore, it is not possible to identify SAOB from coding information in their genome alone. Phylogenetic analysis of ribosomal proteins revealed *Syntrophaceticus* MAG-R2 most closely related to the well characterized SAOB *Syntrophaceticus schinkii* and *Thermacetogenium phaeum* (Fig. 1b). Although the MAG showed only 64% marker gene completeness, it contained the WL pathway except a methylene-tetrahydrofolate (methylene-THF) reductase gene that is missing (Fig. 3b). Moreover, MAG-R2 shared several genes encoding energy conservation mechanisms with *S. schinkii* and *T. phaeum* including the respiratory group 1a [NiFe] hydrogenase that is absent in the other known SAOB [54]. The genomic repertoire for syntrophic acetate oxidation was further identified in *Dethiobacteria* MAG-R7. The 89% complete MAG only lacked the gene for acetate activation, the acetate kinase (Fig. 3b). In the oxidative WL pathway, one ATP has to be invested for acetate activation, and substrate level phosphorylation of methyl-THF to formate would yield one ATP. Thus, there is no net ATP gain and it is still unknown how SAOB conserve energy. To circumvent energy-consuming acetate activation by an acetate kinase to acetyl phosphate, an alternative route via acetaldehyde was recently proposed for *T. phaeum* [55, 56]. *Dethiobacteria* MAG-R7 contained several genes encoding an aldehyde ferredoxin oxidoreductase that could enable this alternative way of acetate activation, or the acetate kinase gene is missed in the assembly. A nearly complete 16S rRNA gene was encoded in MAG-R7 that further confirmed the phylogenetic placement based on ribosomal proteins. Interestingly, MAG-R7 grouped with *Ca*. Syntrophonatronum acetioxidans, a SAOB important in haloalkaline environments [30, 57]. *Ca*. Contubernalis alkalaceticum, another haloalkaliphilic bacterium that was described as SAOB [58], and other syntrophic *Dethiobacteraceae* [57] were closely related to MAG-R7. Together, the phylogeny and the genomic repertoire for syntrophic acetate oxidation suggest the *Dethiobacteria* MAG-R7 as a potential SAOB. Besides carbohydrate fermentation, a complete beta-oxidation pathway was found in MAG-R7 suggesting a high metabolic flexibility potentially including fatty acid degradation (Fig. 1b, Fig. 2).

Mosbæk et al. [59] suggested members of the DTU014 clade (*Firmicutes*) as SAOB. Protein-based stable isotope probing combined with metagenomics identified heavily labeled proteins affiliating with the DTU014 in incubations with ^13^C-labeled acetate although a complete WL pathway was not identified in the respective genome bins [59]. Both DTU014 MAGs obtained in this study (R5 and R6) shared > 99% average nucleotide identity with the MAGs reported by Mosbæk et al*.* [59] and also lack key genes such as the carbon monoxide dehydrogenase/acetyl-CoA synthase complex including the corrinoid iron sulfur protein (Fig. 3b). The oxidative WL pathway is so far the only known route used by SAOB to drive acetate oxidation. However, not all isolated and characterized SAOB encode the complete gene set for this pathway as well. The genomes of four isolated SAOB are completely sequenced, but *Clostridium ultunense* and *Pseudothermotoga lettingae* lack several genes of the WL pathway indicating that other pathways for syntrophic acetate oxidation could exist. Consequently, acetate-oxidizing DTU014 might use such an alternative route. Low energy yield and slow growth rates of syntrophic SCFA-oxidizing bacteria could be a possible explanation that those organisms were often found in low abundances in AD. The high coverage of DTU014 MAG-R5 and MAG-R6 together with their large fraction of 16S rRNA gene fragments (Additional file 1: Table S1, Fig. S2) indicate a high abundance of these species, which contradicts the typical proportions of SAOB in AD communities and rather points to a function different than syntrophic acetate consumption in this biogas reactor.

Other potential syntrophs identified in the metagenome were *Caldatribacterium* (MAG-R9) and *Syntrophomonas* (MAG-R1) species. The putative functions of the poorly characterized candidate phylum *Atribacteria* are as yet little understood. *Atribacteria* associated with AD were proposed as syntrophic propionate-oxidizing bacteria based on metagenomics and transcriptomics [60, 61]; however, genes encoding the methylmalonyl-CoA pathway for propionate oxidation were largely absent in *Caldatribacterium* MAG-R9. Saccharolytic fermentation and syntrophic acetate oxidation via a hypothetical pathway using the glycine cleavage system was also proposed [61, 62]. There is as yet no experimental evidence for syntrophic acetate oxidation among the *Atribacteria*, and physiological inferences are almost exclusively based on metagenome data. MAG-R1 affiliated with cultured *Syntrophomonas* species (Fig. 1b). Like the isolated relatives, *Syntrophomonas* MAG-R1 appears to be specialized for the degradation of fatty acids using the beta-oxidation pathway. MAG-R1 and MAG-R9 did not encode a WL pathway and thus likely did not belong to the acetate-degrading community (Fig. 3b).

### Potential for syntrophic acetate oxidation in enrichment cultures

**Fig. 4:**
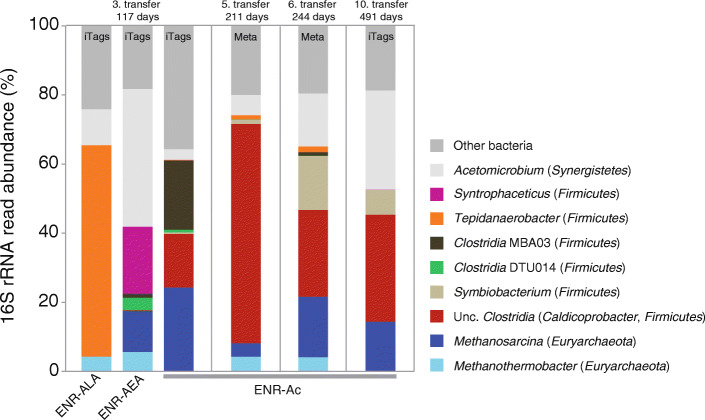
Relative abundance of major bacterial and archaeal groups in acetate-fed enrichment cultures. The microbial community was either analyzed by 16S rRNA gene amplicon sequencing (iTags) or metagenome sequencing (Meta)

Mineral medium containing no electron acceptors other than bicarbonate/CO_2_ was inoculated from a thermophilic biogas reactor (Additional file 1: Table S2). Acetate was repeatedly fed to a concentration of 10 mM. The cultures were incubated at 55 °C and 1–10% was regularly transferred into fresh medium. After 117 days of incubation and three successive transfers, the active cultures were selected for amplicon sequencing to identify the acetate-consuming microbial community (Fig. 4). The enrichments ENR-ALA, ENR-AEA, and ENR-Ac were inoculated from the same reactor but treated slightly different (see “Methods” section for details). Bacteria known as SAOB such as *Syntrophaceticus* and *Tepidanaerobacter* were enriched but also acetoclastic methanogens of the *Methanosarcina* (Fig. 4). In ENR-Ac, *Firmicutes* classified as *Caldicoprobacter* and *Methanosarcina* were enriched whereas known SAOB could not be detected. Active acetate-degrading enrichment cultures with no known SAOB were selected, and cultivation was pursued in the same way (ENR-Ac). The microbial communities were screened again after 211, 244, and 491 days of incubation. *Caldicoprobacter*-related bacteria were enriched from 2% relative abundance in the inoculum up to 63% and were consistently identified as the dominant microbial group in these cultures although their relative abundance was fluctuating (Fig. 4). Even though it is unclear if the detected *Methanosarcina* performed acetoclastic or hydrogenotrophic methanogenesis, the presence of strictly hydrogenotrophic *Methanothermobacter* strongly suggest syntrophic acetate oxidation. In addition, uncultured *Anaerolineaceae* (*Chloroflexi*), *Symbiobacterium*, and *Acetomicrobium* were also enriched in the culture ENR-Ac (Fig. 4; Additional file 1: Fig. S3). Uncultured *Anaerolineaceae* were previously linked to homoacetogenesis in AD using a combined metagenomics and transcriptomics approach [63]. The high acetate concentrations fed to the cultures and phases of starvation were stress factors for the microbes. Such phases of imbalance might allow the coexistence of homoacetogenic bacteria, SAOB, acetoclastic, and hydrogenotrophic methanogens. *Acetomicrobium* was identified in all cultures and was often among the most abundant. The partial 16S rRNA sequence of the dominant OTU (~ 400 bp length) from the enrichment cultures was closely related to *A. hydrogeniformans* (> 99% identity), which was described to ferment simple sugars and amino acids [62]. We tested all three related species (*A. hydrogeniformans* DSM22491, *A. thermoterrenum* DSM13490, and *A. mobile* DSM13181) with both *Methanoculleus thermophilus* DSM2373 and *Methanothermobacter thermoautotrophicus* DSM1053 in a defined co-culture for syntrophic growth on acetate. Moreover, the *Acetomicrobium* sp. purified from our enrichment cultures was also tested for growth with both methanogens. Under the applied conditions, no acetate consumption or methane formation was observed for any of the defined co-cultures. Interestingly, *Acetomicrobium* was also enriched in thermophilic propionate-degrading enrichment cultures inoculated from the same biogas reactor [64]; however, their role in these syntrophic communities still remains enigmatic.

Enrichment ENR-Ac was further sampled at day 244 for metagenome sequencing. Two largely complete MAGs with less than 2% contamination (Additional file 1: Table S1) that affiliated with *Firmicutes* were recovered from the metagenome. Both MAGs did not contain 16S rRNA gene fragments that could be used for phylogenetic identification. Based on ribosomal proteins, MAG-E1 was distantly related to *Caldicoprobacter* sp., whereas MAG-E2 was related to *Calderihabitans maritimus* (Fig. 1b). Amplicon sequencing revealed that the clade assigned to *Caldicoprobacter* was dominated by two major OTUs at day 491. Together with the coverage information (Additional file 1: Table S1), this suggest that the MAGs E1 and E2 represented the dominant species of the *Caldicoprobacter* clade. *Firmicutes* MAG-E1 that showed 97.6% marker gene completeness lacked central genes of the WL pathway whereas a complete WL pathway could be identified in MAG-E2 (Fig. 3b). A comparison of their genomic repertoire revealed that the metabolism of MAG-E2 is more versatile (Figs. 1b, 2, 3b). Central genes of butyrate and propionate metabolism, carbohydrate fermentation, beta oxidation, and amino acid metabolism were detected. Moreover, a respiratory complex I, genes for a dissimilatory sulfite reductase (DsrAB) together with DsrCJKMOP as well as a truncated denitrification pathway including nitrate reductase, nitrite reductase, and nitric-oxide reductase were encoded in the genome (Additional file 1: Fig. S4, Fig. S5). This strongly suggests that MAG-E2 can utilize various electron acceptors. The MAG-E2 contained genes encoding a NAD-dependent FDH and a FDH complex (Fig. 3b) that is likely membrane associated and periplasmatically oriented as indicated by a TAT (twin-arginine translocation) signal peptide as well as transmembrane helices. The structure of this complex is comparable to the membrane-bound FDH of *Thermacetogenium phaeum*, which is only expressed during syntrophic growth on acetate [55]. Reverse electron transport could be driven in a similar manner to that of *Syntrophomonas wolfei* when growing syntrophically on butyrate [65–67]. Electrons may be shuttled by an electron transfer flavoprotein (ETF) to an ETF-quinone oxidoreductase that reduces the quinone pool. The periplasmatically oriented FDH complex could then reoxidize the reduced quinone pool. MAG-E2 further encodes an electron confurcating FeFe-hydrogenase group A3 (Ech) and a heterodisulfide reductase (Hdr) complex that is possibly involved in reverse electron transport (Additional file 1: Fig. S4). Stable isotope probing further suggested *Caldicoprobacter*-related *Firmicutes* as acetate-oxidizing bacteria in thermophilic methanogenic communities fed with ^13^C-labeled acetate [68]. The major fraction of the heavily labeled bacterial DNA was assigned to the *Caldicoprobacter*-related organisms [68]. SAOB are fastidious and slow growing bacteria that are difficult to culture. So far, no SAOB were isolated together with hydrogenotrophic methanogens from our enrichment cultures. Thus, additional research is indispensable to confirm syntrophic acetate metabolism among these uncultured bacteria and to unravel their mechanism for syntrophy.

### Amplicon sequencing revealed stable bacterial and archaeal communities in thermophilic dry-fermentation of biowaste

To examine if the identified putatively SAOB were a regular component of the microbial community in thermophilic solid-state digestion of biowaste, we selected nine full-scale anaerobic reactors located in Germany and the Netherlands for 16S rRNA gene amplicon sequencing (iTags) of the V3-V4 region (> 400 bp). The reactor that was used for metagenomic analysis was also included for the amplicon sequencing approach (PFL9). The combination of amplicon sequencing and metagenomics enabled us to link the metabolic insights to a stable core population thus allowing more general implications and a broader perspective on microbial interactions in thermophilic dry-fermentation of biowaste.

**Fig. 5:**
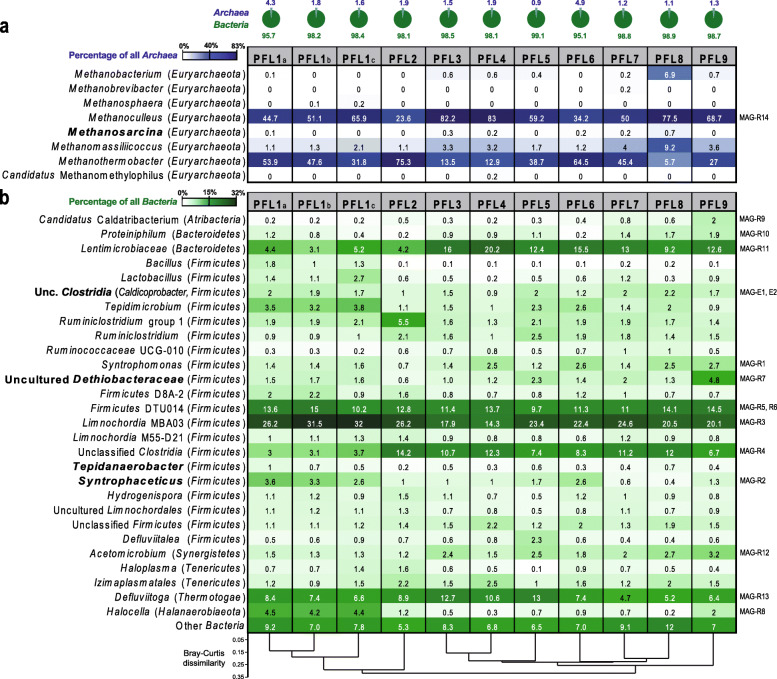
Microbial community composition in nine full-scale thermophilic biowaste digesters. The archaeal fraction of total prokaryotes and the relative abundance of archaeal amplicons was revealed by 16S rRNA gene amplicon sequencing (**a**). Percentages of taxa relative to total bacterial amplicons are only shown for major groups that contributed ≥ 1% to all bacterial 16S rRNA sequences in at least one sample (**b**). Bray-Curtis dissimilarity based on OTUs clustered at 97% identity is depicted at the bottom

All sampled biogas reactors treat biowaste in dry fermentation (approximately 30% dry matter content) and were operated between 54 and 55 °C. More than 550,000 quality trimmed amplicon reads were kept for phylogenetic classification (Additional file 1: Table S3). *Archaea* usually contributed 1–2% to total prokaryotic iTags (Fig. 5a), which reflects the importance of low abundant but highly active microorganisms for the functionality of a community. Only in one reactor (PFL6), the archaeal 16S rRNA gene abundance reached 4.9%, and *Archaea* were also enriched in the separated liquid fraction (PLF1a) of reactor PFL1. The archaeal fraction of total prokaryotes is probably slightly underestimated as the average 16S rRNA gene copy number per genome for *Bacteria* (*n* = 5) is higher than those of the dominant methanogens (*Methanothermobacter*, *n* = 2; *Methanoculleus*, *n* = 1). *Methanoculleus*, *Methanothermobacter*, and *Methanomassiliicoccus* were ubiquitous throughout the sampled biogas reactors with the first two genera predominating the archaeal community. Together, *Methanoculleus* and *Methanothermobacter* contributed 83.2–98.9% to archaeal iTags (Fig. 5a). In nearly half of the systems (4 out of 9), acetoclastic methanogens of the genera *Methanosaeta* and *Methanosarcina* were not detected using amplicon sequencing (Fig. 5a). In five reactors, *Methanosarcina* accounted for 0.1–0.7% of *Archaea*, which is equivalent to a maximum of 0.008% of the total prokaryotic iTags. This very low relative abundance is contradicting to studies that identified *Methanosarcinales* as abundant core members of the archaeal community in AD [4, 6, 7, 44]. More quantitative methods such as fluorescence in situ hybridization will be necessary to estimate exact *Bacteria* to *Archaea* ratios; however, the dominance of strictly hydrogenotrophic over acetoclastic methanogens within the archaeal community points to a favorable opportunity for SAOB in these AD systems.

Like for the *Archaea,* the bacterial community showed a recurring diversity pattern among the biogas reactors (Fig. 5b). Alpha diversity measures further indicate a low diversity within the digesters (Additional file 1: Table S4). This is in accordance with the findings that thermophilic systems harbor a lower number of species compared to mesophilic reactors [69, 70]. In contrast, the degradation of complex substrates was previously linked to a high diversity whereas a smaller number of species was identified in reactors that degrade simple substrates [44]. Overall, the microbial community identified by amplicon sequencing was in good coherence with the outcomes of the metagenomic approach for PFL9 (Figs. 1a and 5), and the identified MAGs adequately covered the major groups of the microbial community.

*Firmicutes*, *Bacteroidetes,* and *Thermotogae* were consistently identified as the dominant clades on phylum level in accordance with previous findings that reported a high abundance of those three phyla in thermophilic and biowaste-treating digesters [7, 44]. Together, they accounted for 89.6–95.2% of the bacterial community (Additional file 1: Fig. S6). Among the *Firmicutes*, a large fraction of reads affiliated with uncultured bacteria of the MBA03 and the DTU014 clade. Together, they contributed 28–46.5% to all bacterial iTags and make up more than half of the *Firmicutes* (Fig. 5b). The MBA03 and DTU014 groups cluster sequences of uncultured bacteria at order to family level. However, the MBA03 clade was represented only by one single OTU that accounted for more than 97% of the reads, and the DTU014 clade comprised two OTUs that contributed 92.3–96.6% of their iTags revealing very low species diversity within these groups. Despite their abundance and the identification as members of the core microbiome in mesophilic and thermophilic AD, their metabolic potential and physiology is still poorly characterized [6, 71]. Together with the metagenomic analysis, this suggests that few MBA03 and DTU014 species are central to carbohydrate fermentation throughout these thermophilic AD reactors. As most of the MAGs from the metagenome did not contain 16S rRNA gene fragments, they could not directly linked to the OTUs or clustered groups. Instead, the phylogenetic position based on concatenated ribosomal proteins, the taxonomic classification of all coding sequences, and coverage information were used for the assignment (Fig. 5).

Unlike the *Archaea*, potential acetate-consuming bacteria of the genera *Syntrophaceticus* and *Tepidanaerobacter* were consistently identified contributing 0.4–3.6% and 0.2–1% to the bacterial community, respectively. *Syntrophaceticus schinkii* was described as a mesophilic species operating at a temperature range from 25 to 40 °C [29]; however, thermophilic representatives of the *Syntrophaceticus* were recently identified as drivers of syntrophic acetate oxidation in acetate-fed chemostats [72] suggesting a wide temperature range for acetate degradation within this genus. Uncultured *Dethiobacteraceae* were consistently identified with similar abundances like the other SPOB but exceptionally high in abundance (~ 5%) in PFL9, the reactor used for our metagenome survey. Moreover, iTags affiliating with *Caldicoprobacter* always contributed more than 1% to total bacterial amplicons (Fig. 5b). The OTUs identified in the enrichment cultures were also among the dominant *Caldicoprobacter* OTUs in the biogas reactors. The OTUs only share approximately 91% sequence identity to isolated *Caldicoprobacter* spp. thus indicating a phylogenetic and physiological differentiation between these bacteria. Taken together, uncultured *Dethiobacteraceae* and unclassified *Clostridia* distantly related to *Caldicoprobacter* might harbor novel putatively syntrophic acetate-consuming bacteria that are widespread in thermophilic dry fermentation of biowaste. The reactors harbor a diverse acetate-consuming community impressively dominated by SAOB over acetoclastic methanogens.

### Ecological considerations for syntrophic acetate metabolism in thermophilic AD of biowaste

Despite the fact that hydrogenotrophic and acetoclastic methanogens generally coexist, it has been shown by radioisotope probing combined with metagenomic sequencing that the acetoclastic pathway for methanogenesis can be dominant while hydrogenotrophic methanogens showed higher abundances [73]. However, the absence or clear minority of *Methanosarcina* and other acetoclastic methanogens but ubiquity of diverse potential SAOBs on the other hand strongly suggests that acetate is primarily oxidized by bacteria in the studied biogas reactors. Digesters that treat manure or protein-rich material such as biowaste can have high levels of total ammonia nitrogen (TAN) above 4 g NH_4_^+^-N/kg (e.g., [7, 45, 73]) and ammonia inhibition is a major issue in AD (reviewed by [74]). It has been demonstrated that a thermophilic community in AD of biowaste can tolerate significantly more free ammonia compared to a mesophilic community [15]. This was also shown specifically for the hydrogenotrophic methanogens [26]. The toxic-free ammonia increases with elevating temperature and pH, and its concentration was suggested as major factor that determines the assemblage of the acetate-consuming bacterial and archaeal community [17, 19, 22, 24, 25, 44]. TAN concentrations in reactor PFL1 and PFL8 have been regularly measured and usually ranged between 1.7 and 3 g NH_4_^+^-N/kg (Additional file 1: Fig. S7) and were in the lower range for AD of biowaste (e.g., [7, 45]). The composition of biowaste varies over time thus fluctuations of TAN concentrations are expected. However, the community in reactor PFL1 was very similar when sampled at different time points (PFL1b and PFL1c, Additional file 1: Table S2). Although it requires additional research to unravel the environmental factors that shape this consistent community, it appears that dry matter content, temperature, and the substrate were more important than the TAN level alone. Interestingly, such adapted community towards syntrophic acetate oxidation might be more tolerant to ammonia fluctuations caused by high protein loadings and consequently more stable with respect to process failure due to ammonia inhibition. In AD, a major fraction of the carbon flow to methane occurs via acetate. A better understanding of SPOB will be important and could provide a basis for the management of microbial AD communities.

## Conclusions

By combining metagenomics with 16S rRNA gene amplicon sequencing and enrichment cultivation, this study allowed us to link the identified organisms with their potential function in the AD food web. The metabolic reconstruction of metagenome assembled genomes from a full-scale biogas reactor and enrichment cultures identified novel putatively acetate-oxidizing bacteria with high metabolic plasticity. The unique ability to reverse the WL pathway or likely using other pathways for syntrophic acetate oxidation is key for SAOB to compete with the carbohydrate-fermenting majority. Beyond the genomic evidence, syntrophic acetate oxidation was demonstrated by the enrichment of *Syntrophaceticus*, *Tepidanaerobacter*, and *Clostridia* distantly related to *Caldicoprobacter.* Acetate-dependent catabolic processes in the biogas reactors were largely attributed to bacteria as acetoclastic methanogens were completely outnumbered by the SAOB. Thermophilic dry-fermentation of biowaste possibly provides an exceptional ecosystem to study SAOB and for the enrichment and isolation of novel SAOB. Assuming that the identified SAOB are actually active as acetate-oxidizers, this underpins the importance of these bacteria for process functioning and overall organic carbon mineralization. Further studies applying proteomics or transcriptomics and isotope labeling techniques will be crucial to determine their in situ activity and to quantify syntrophic acetate oxidation versus acetoclastic methanogenesis.

## Methods

### Sampling and enrichment of syntrophic acetate-oxidizing bacteria

Samples were taken from nine thermophilic full-scale biogas plants (Additional file 1: Table S2). All samples for DNA extraction were stored at − 20 °C immediately after sampling. Subsamples taken from the reactor PFL5 were suspended in mineral medium as described recently [64]. Acetate was repeatedly fed from anoxic sterile stock solutions to a final concentration of 5–10 mM. If necessary, the pH was corrected to approximately 7.5 with NaOH from an anoxic sterile stock solution (1 M). Enrichments were carried out over a period of 491 days by successive transfers (*n* = 10) of 1–10% into fresh medium. ENR-ALA was once fed with 10 mM lactic acid, and ENR-AEA was once fed with 10 mM ethanol before the third transfer. Both treatments were further on fed with acetate again. Enrichment ENR-Ac was solely fed with acetate. *Acetomicrobium hydrogeniformans* DSM22491, *A. thermoterrenum* DSM13490, *A. mobile* DSM13181, *Methanoculleus thermophilus* DSM2373, and *Methanothermobacter thermoautotrophicus* DSM1053 were purchased from the Leibniz Institute DSMZ-German Collection of Microorganisms and Cell Cultures and cultured according to their recommendations. To determine acetate oxidation, methane and SCFAs were measured with a gas chromatograph as described previously [64].

### Metagenome sequencing and analysis

DNA was extracted using the PowerViral DNA/RNA kit (Quiagen, Hilden, Germany) according to manufacturer’s instructions. Libraries were prepared using the Nextera XT kit (Illumina Inc., CA, USA) according to manufacturer’s protocol and paired end sequenced (2x 300 bp) on MiSeq platform using V3 chemistry (Illumina Inc., CA, USA). All bioinformatics is described in detail in Additional file 2: Supplementary Methods. In brief, all tools from raw read processing to phylogenetic analysis of final MAGs were embedded and automated in a Snakemake [40] workflow. The parameters and settings of the software applied in this study can be further found in our Snakefile (https://www.knutt.org/Snakefiles). In the first step, quality trimmed reads were used for taxonomic and functional classification followed by assembly and binning based on tetra-nucleotide frequencies and coverage. The annotation was mainly performed with MetaErg [75]; however, further annotation tools and custom databases that we considered as useful were included in the pipeline. Concatenated ribosomal proteins were used to determine the phylogenetic position of the MAGs. The automated metagenome analysis pipeline is available at github.com/KnuttPipeline.

### 16S rRNA gene amplicon sequencing

The bacterial and archaeal diversity in the biogas reactors was determined by analyzing the V3-V4 region of the 16S rRNA gene using Illumina MiSeq sequencing of barcoded amplicons. Amplicons were prepared using primers Pro341f/Pro805r [76] targeting the domains *Bacteria* and *Archaea*. PCR conditions were as described earlier [64]. PCR products were purified using AMPure XP beads (Beckman Coulter), and libraries were prepared using the Nextera XT kit (Illumina Inc., CA, USA) according to manufacturer’s protocol. Libraries were quantified with a Qubit 3.0 fluorimeter (Thermo Fisher Scientific Inc., USA) and paired end sequenced (2x 300 bp) on MiSeq platform using V3 chemistry (Illumina Inc., CA, USA). Remaining Nextera XT adapter as well as primer sequences were removed, and the reads were trimmed at quality score Q20 using cutadapt [77]. The reads were merged with BBMerge [78] and OTUs clustered at 97% identity were generated with VSEARCH [79]. The OTUs were classified using SINA and the SILVA database SSU Ref NR r132 [80, 81]. Alpha and beta diversity measures were calculated using phyloseq [82] or PAST [83].

## Supplementary information

**Additional file 1: Figure S1.** Relative abundance of hydrogenase classes identified in the metagenome. **Figure S2.** Relative abundance of metagenomic 16S rRNA gene fragments from PFL9 affiliating with *Bacteria* and *Archaea* as shown in Fig. 1a but the highest classification rank of the major groups is depicted. **Figure S3.** Relative abundance of bacterial and archaeal groups that contributed ≥1% to total 16S rRNA sequences in at least one sample in acetate-fed enrichment cultures. The microbial community was either analyzed by 16S rRNA gene amplicon sequencing (iTags) or metagenome sequencing (Meta). **Figure S4.** A simplified overview of the metabolic functions reconstructed from *Firmicutes* MAG-E2. Hyd, [FeFe] hydrogenase group A3; Hdr, heterodisulfide reductase; Rnf, Na^+^/H^+^ translocating ferredoxin:NAD^+^ oxidoreductase; Q, quinone; ETF, electron transfer flavoprotein; ETF-Q OR, electron transfer flavoprotein/quinone oxidoreductase; FDH, formate dehydrogenase; Dsr, dissimilatory sulfite reductase; C, DsrC; Nar, nitrate reductase; Nir, nitrite reductase; Nor, nitric oxide reductase; SDH, succinate dehydrogenase; GH, glycosyl hydrolase; EM, electron mediator; CM, cytoplasmic membrane. **Figure S5.** Maximum Likelihood (RAxML) tree of dissimilatory sulfite reductase genes (DsrAB) recovered from *Firmicutes* MAG-E2. Closed circles indicate bootstrap support ≥70%. The reference database used for phylogenetic reconstruction contained 340 sequences of DsrAB proteins (Loy et al., 2008; *Environ. Microbiol*. 11: 289-299). **Figure S6.** Microbial community composition in nine full-scale thermophilic biowaste digesters summarized at phylum level. The relative abundance was revealed by 16S rRNA gene amplicon sequencing. **Figure S7.** Total ammonia nitrogen (g NH_4_^+^-N/kg) and pH in PFL1 and PFL8. **Table S1.** General statistics about MAGs recovered from the reactor PFL9 and from the enrichment culture. **Table S2.** Overview of the sampled biogas reactors and performed experiments. **Table S3.** Number of quality trimmed 16S rRNA gene amplicons kept for taxonomic classification. **Table S4.** Comparison of alpha diversity measures determined for the nine biogas reactors.

**Additional file 2.** Supplementary Methods.

## Data Availability

All nucleotide sequences obtained in this study have been deposited in GenBank. Amplicon sequences from the 16S rRNA gene survey were deposited in NCBI BioProject PRJNA607872 and metagenome reads are available in BioProject PRJNA607871. MAGs are available on https://www.knutt.org/.
