# Supplementary material for: Syntrophic acetate oxidation replaces acetoclastic methanogenesis during thermophilic digestion of biowaste
Source: Microbiome. 2020 Jul 3;8:105. doi: 10.1186/s40168-020-00862-5 (PMC7334858; doi:10.1186/s40168-020-00862-5)
Supplement: Supplementary file 2 — Additional file 1: Figure S1. Relative abundance of hydrogenase classes identified in the metagenome. Figure S2. Relative abundance of metagenomic 16S rRNA gene fragments from PFL9 affiliating with Bacteria and Archaea as shown in Fig. 1a but the highest classification rank of the major groups is depicted. Figure S3. Relative abundance of bacterial and archaeal groups that contributed ≥1% to total 16S rRNA sequences in at least one sample in acetate-fed enrichment cultures. The microbial community was either analyzed by 16S rRNA gene amplicon sequencing (iTags) or metagenome sequencing (Meta). Figure S4. A simplified overview of the metabolic functions reconstructed from Firmicutes MAG-E2. Hyd, [FeFe] hydrogenase group A3; Hdr, heterodisulfide reductase; Rnf, Na+/H+ translocating ferredoxin:NAD+ oxidoreductase; Q, quinone; ETF, electron transfer flavoprotein; ETF-Q OR, electron transfer flavoprotein/quinone oxidoreductase; FDH, formate dehydrogenase; Dsr, dissimilatory sulfite reductase; C, DsrC; Nar, nitrate reductase; Nir, nitrite reductase; Nor, nitric oxide reductase; SDH, succinate dehydrogenase; GH, glycosyl hydrolase; EM, electron mediator; CM, cytoplasmic membrane. Figure S5. Maximum Likelihood (RAxML) tree of dissimilatory sulfite reductase genes (DsrAB) recovered from Firmicutes MAG-E2. Closed circles indicate bootstrap support ≥70%. The reference database used for phylogenetic reconstruction contained 340 sequences of DsrAB proteins (Loy et al., 2008; Environ. Microbiol. 11: 289-299). Figure S6. Microbial community composition in nine full-scale thermophilic biowaste digesters summarized at phylum level. The relative abundance was revealed by 16S rRNA gene amplicon sequencing. Figure S7. Total ammonia nitrogen (g NH4+-N/kg) and pH in PFL1 and PFL8. Table S1. General statistics about MAGs recovered from the reactor PFL9 and from the enrichment culture. Table S2. Overview of the sampled biogas reactors and performed experiments. Table S3. Number of quality [file 40168_2020_862_MOESM1_ESM.pdf]

Additional file 1 – Supplementary Figures and Tables

Syntrophic acetate oxidation replaces acetoclastic methanogenesis during thermophilic digestion of biowaste

Stefan Dyksma*, Lukas Jansen and Claudia Gallert

Faculty of Technology, Microbiology – Biotechnology, University of Applied Sciences Emden/Leer, Emden, Germany

* To whom correspondence should be addressed: Faculty of Technology, Microbiology – Biotechnology, University of Applied Sciences Emden/Leer, Emden, Germany

Tel +49 4921 807 1483, stefan.dyksma@hs-emden-leer.de

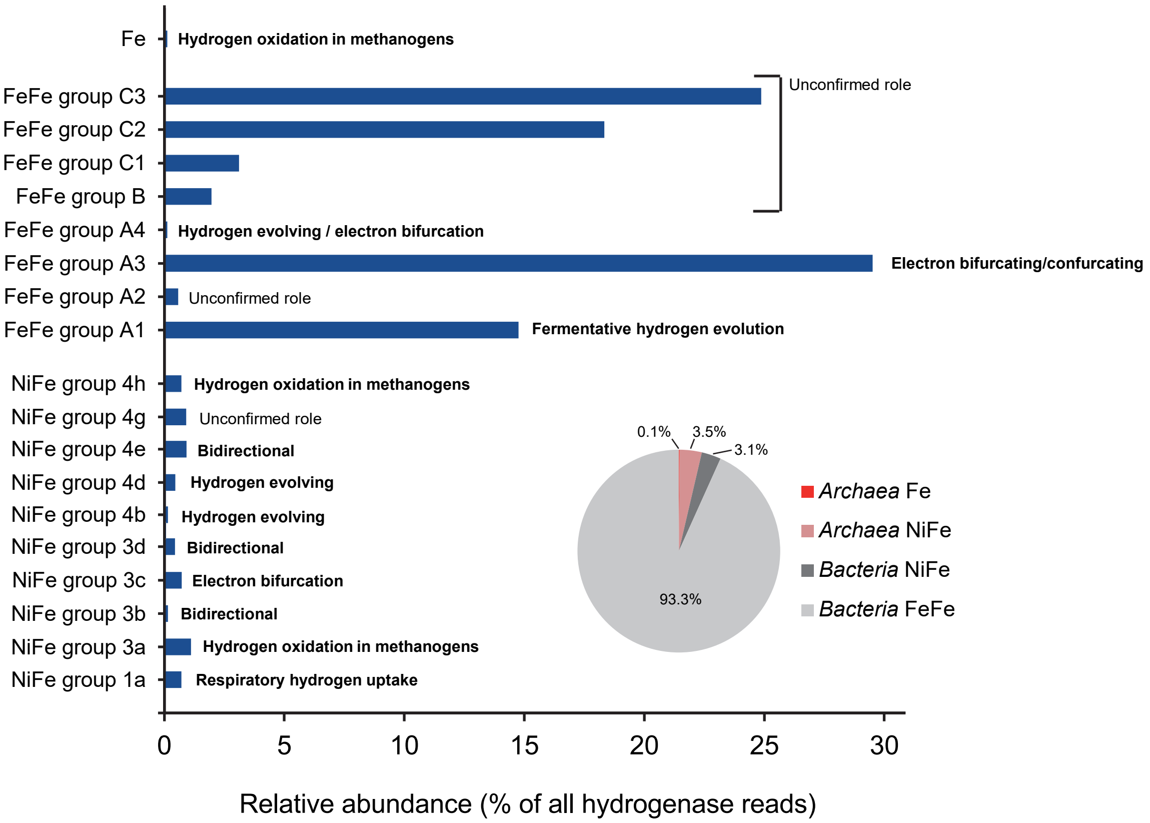

Figure S1. Relative abundance of hydrogenase classes identified in the metagenome.

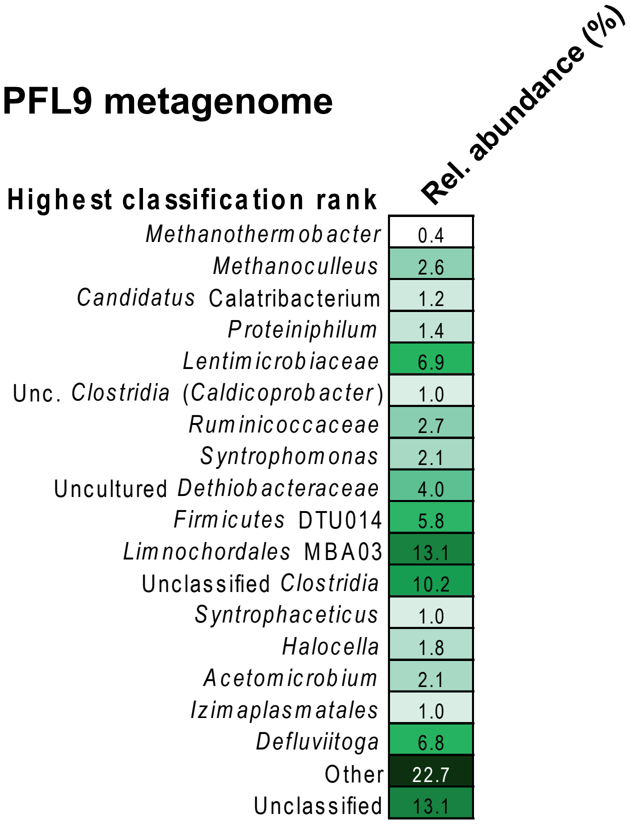

Figure S2. Relative abundance of metagenomic 16S rRNA gene fragments from PFL9 affiliating with *Bacteria* and *Archaea* as shown in Fig. 1a but the highest classification rank of the major groups is depicted.

	ALA	AEA	ENR-Ac			
	117 d	117 d	117 d	211 d	244 d	491 d
Methanothermobacter (*Euryarchaeota*)	4.3	5.7	0	4.3	4.2	0.1
***Methanosarcina* (*Euryarchaeota*)**	0	11.8	24.3	3.9	17.4	14.4
Candidatus Caldatribacterium (*Atribacteria*)	0	0	1.1	0	0	0
Lentimicrobiaceae (*Bacteroidetes*)	0	2.1	8	0	0	0
Anaerolinea (*Chloroflexi*)	0.4	0	1.9	0	0	0
Uncultured *Anaerolineaceae* (*Chloroflexi*)	0	4.3	1.9	1	0.6	14.6
Anaerolineae SBR1031 (*Chloroflexi*)	0	0.2	0.9	0.3	2.2	0
Candidatus Syntrophosphaera (*Cloacimonetes*)	0.6	2.5	0	0	0	0
Coprothermobacter (*Coprothermobacteraeota*)	9.2	0	0.2	0	0	0
Bacillus (*Firmicutes*)	0	0	1.1	0	0	0
Candidatus Desulforudis (*Firmicutes*)	0	0	2.4	1.7	0	0
**Unc. *Clostridia* (*Caldicoprobacter*, *Firmicutes*)**	0	0.2	15.5	63.4	25.2	31
Symbiobacterium (*Firmicutes*)	0	0	0.4	1.3	15.6	7.2
Desulfitibacter (*Firmicutes*)	0.1	4.3	2.8	5.3	6.1	1.3
Desulfotomaculum (*Firmicutes*)	8.2	0.7	4.1	2.6	0.2	0.9
Acetivibrio (*Firmicutes*)	0	0	4.3	0	0	0
Syntrophomonas (*Firmicutes*)	0	0	0	6.3	6.1	0.1
Firmicutes DTU014 (*Firmicutes*)	0	3.6	0.9	0	0.1	0
Clostridia M55-D21 (*Firmicutes*)	0	0	1.5	0	0	0
Limnochordia MBA03 (*Firmicutes*)	0	1.1	20.0	0	1	0
***Tepidanaerobacter* (*Firmicutes*)**	61.2	0	0.2	1.2	1.6	0
Unclassified *Thermoanaerobacteraceae* (*Firmicutes*)	0	2.5	0	0	0	0
***Syntrophaceticus* (*Firmicutes*)**	0	19.4	0	0	0	0
Uncultured *Thermoanaerobacteraceae* (*Firmicutes*)	0	0	0	0	2.9	0
Pseudomonas (*Proteobacteria*)	0	0	0	1.7	0	1.3
Acetomicrobium (*Synergistetes*)	10.4	39.9	3	5.9	15.3	28.7
Defluviitoga (*Thermotogae*)	4.9	0.2	1.7	0	0	0
	iTags	iTags	iTags	Meta	Meta	iTags

Figure S3. Relative abundance of bacterial and archaeal groups that contributed ≥1% to total 16S rRNA sequences in at least one sample in acetate-fed enrichment cultures. The microbial community was either analyzed by 16S rRNA gene amplicon sequencing (iTags) or metagenome sequencing (Meta).

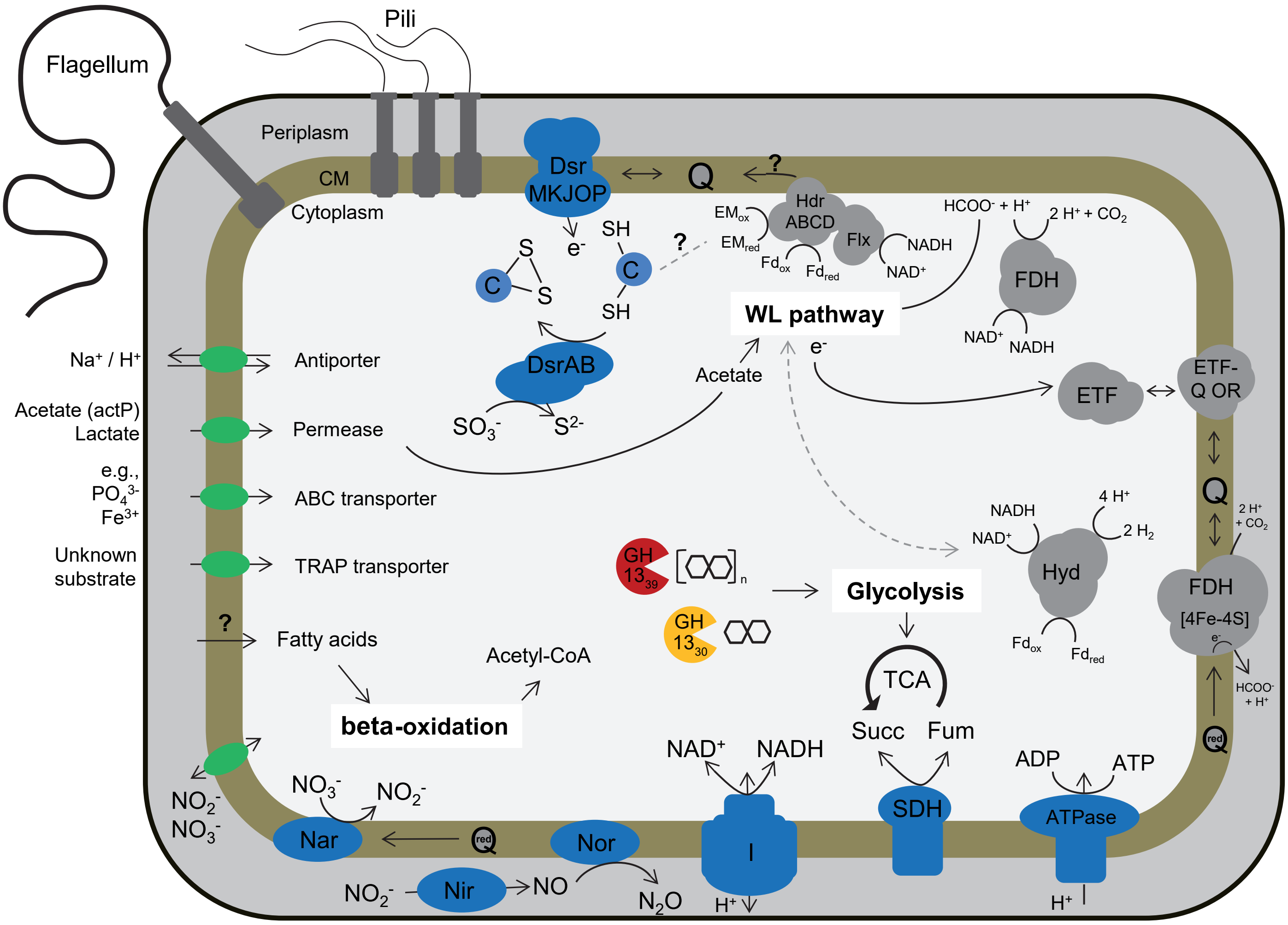

Figure S4. A simplified overview of the metabolic functions reconstructed from *Firmicutes* MAG-E2. Hyd, [FeFe] hydrogenase group A3; Hdr, heterodisulfide reductase; Rnf, Na^+/H^+ translocating ferredoxin:NAD^+ oxidoreductase; Q, quinone; ETF, electron transfer flavoprotein; ETF-Q OR, electron transfer flavoprotein/quinone oxidoreductase; FDH, formate dehydrogenase; Dsr, dissimilatory sulfite reductase; C, DsrC; Nar, nitrate reductase; Nir, nitrite reductase; Nor, nitric oxide reductase; SDH, succinate dehydrogenase; GH, glycosyl hydrolase; EM, electron mediator; CM, cytoplasmic membrane.

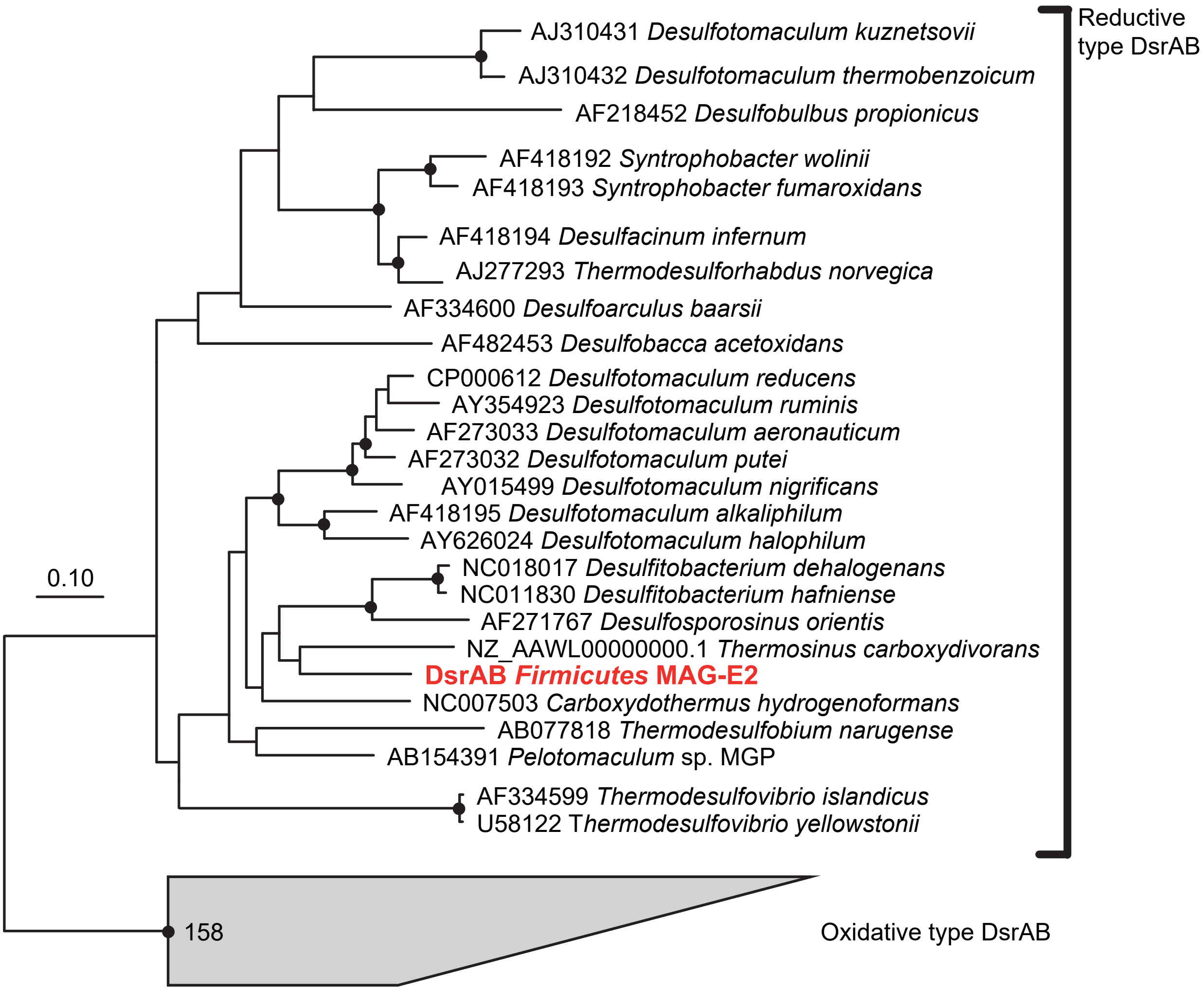

Figure S5. Maximum Likelihood (RAxML) tree of dissimilatory sulfite reductase genes (DsrAB) recovered from *Firmicutes* MAG-E2. Closed circles indicate bootstrap support ≥70%. The reference database used for phylogenetic reconstruction contained 340 sequences of DsrAB proteins (Loy et al., 2008; *Environ. Microbiol.* 11: 289-299).

	PFL1[a]	PFL1[b]	PFL1[c]	PFL2	PFL3	PFL4	PFL5	PFL6	PFL7	PFL8	PFL9
Actinobacteria	0.9	0.7	0.9	0.2	0.2	0.2	0.1	0.3	0.7	0.3	0.3
Atribacteria	0.2	0.2	0.2	0.5	0.3	0.2	0.3	0.4	0.8	0.6	2
Bacteroidetes	5.8	3.9	5.6	4.4	16.8	21.1	13.5	15.6	14.4	11.4	14.5
Firmicutes	75.7	79.9	77.3	79.3	64.6	62.7	68.6	70.7	74.2	76.1	69.3
Halanaerobiaeota	4.5	4.2	4.4	1.2	0.5	0.3	0.7	0.9	0.7	0.2	2
Proteobacteria	0.6	0.2	0.6	0.4	0.2	0.2	0.1	0.1	0.5	0.3	0.2
Synergistetes	1.5	1.3	1.3	1.2	2.5	1.6	2.5	1.8	2	2.9	3.3
Tenericutes	2	1.7	2.9	3.8	2.1	3	1.1	2.6	1.9	2.9	1.9
Thermotogae	8.4	7.4	6.6	8.9	12.7	10.6	13	7.4	4.7	5.2	6.4
Other phyla	0.5	0.5	0.2	0.1	0.1	0.1	0.1	0.2	0.1	0.1	0.1

Figure S6. Microbial community composition in nine full-scale thermophilic biowaste digesters summarized at phylum level. The relative abundance was revealed by 16S rRNA gene amplicon sequencing.

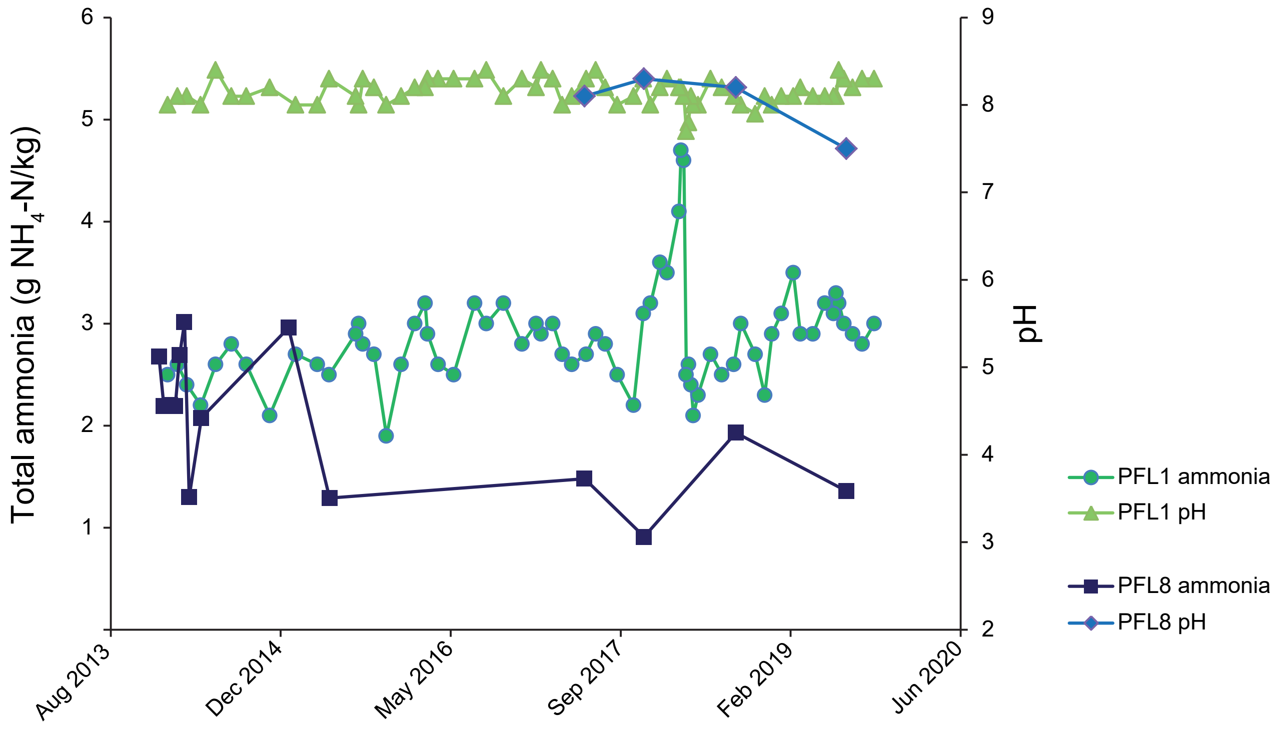

Figure S7. Total ammonia nitrogen (g NH_4^+-N/kg) and pH in PFL1 and PFL8.

Table S1. General statistics about MAGs recovered from the reactor PFL9 and from the enrichment culture. Overall, 57% of the reads from the reactor metagenome were aligned to the 14 MAGs R1-R14 and 28% of the reads from the enrichment metagenome were aligned to the MAGs E1 and E2.

MAG		Completeness (%)	Contamination (%)	Coverage (x fold)	Phylum
Syntrophomonas	MAG-R1	95.8	0.9	6.8	*Firmicutes*
Syntrophaceticus	MAG-R2	63.9	0.3	4.1	*Firmicutes*
Limnochordia	MAG-R3	94.9	3.0	29.9	*Firmicutes*
Limnochordia	MAG-R4	94.2	1.9	13.7	*Firmicutes*
Firmicutes DTU014	MAG-R5	94.1	1.0	15.1	*Firmicutes*
Firmicutes DTU014	MAG-R6	87.8	0.5	23.1	*Firmicutes*
Dethiobacteria	MAG-R7	89.4	1.3	15.0	*Firmicutes*
Halanaerobiaceae	MAG-R8	67.1	0.0	4.9	*Halanaerobiaeota*
Caldatribacterium	MAG-R9	89.0	0.2	6.1	*Atribacteria*
Dysgonomonadaceae	MAG-R10	61.5	0.3	5.1	*Bacteroidetes*
Sphingobacteriales	MAG-R11	72.1	1.1	15.8	*Bacteroidetes*
Acetomicrobium	MAG-R12	88.1	3.4	8.1	*Synergistetes*
Defluviitoga	MAG-R13	88.8	14.3	17.0	*Thermotogae*
Methanoculleus	MAG-R14	99.5	97.2	10.3	*Euryarchaeota*
Firmicutes	MAG-E1	97.6	0.0	96.9	*Firmicutes*
Firmicutes	MAG-E2	92.2	1.3	53.5	*Firmicutes*

Table S2. Overview of the sampled biogas reactors and performed experiments.

Reactor	Fraction	Sampling date	Performed experiments
PFL1a	liquid	2018-11-15	Amplicon sequencing
PFL1b	not separated	2018-11-15	Amplicon sequencing
PFL1c	not separated	2017-10-10	Amplicon sequencing
PFL2	not separated	2017-11-08	Amplicon sequencing
PFL3	not separated	2017-11-03	Amplicon sequencing
PFL4	not separated	2017-11-08	Amplicon sequencing
PFL5	not separated	2017-10-05	Amplicon sequencing, enrichment cultures
PFL6	not separated	2017-11-08	Amplicon sequencing
PFL7	not separated	2017-11-07	Amplicon sequencing
PFL8	not separated	2017-11-07	Amplicon sequencing
PFL9	not separated	2017-11-07	Amplicon sequencing, metagenomics

Table S3. Number of quality trimmed 16S rRNA gene amplicons kept for taxonomic classification.

Sample	No. of 16S rRNA gene amplicons kept for classification
PFL1a	97,661
PFL1b	87,823
PFL1c	50,743
PFL2	38,255
PFL3	42,643
PFL4	57,443
PFL5	27,569
PFL6	25,705
PFL7	40,807
PFL8	36,606
PFL9	45,188

Table S4. Comparison of alpha diversity measures determined for the nine biogas reactors.

Measure	Reactor PFL										
	1a	1b	1c	2	3	4	5	6	7	8	9
No. $OTUs_{0.97}$	1169	1050	969	793	829	871	822	742	918	959	829
Subsampled (n) reads	22124										
Simpson	0.93	0.90	0.90	0.91	0.93	0.94	0.92	0.94	0.93	0.94	0.94
Shannon	4.06	3.77	3.86	3.59	3.81	3.75	3.73	3.86	3.93	4.07	3.90
Evenness	0.05	0.04	0.05	0.05	0.05	0.05	0.05	0.06	0.06	0.06	0.06
Fisher	263.1	229.3	207	160.8	170	180.9	168.2	148	193.3	204.3	170
Chao-1	2110	1982	1638	1434	1469	1516	1248	1062	1393	1576	1285
