# Supplementary material for: Syntrophic acetate oxidation replaces acetoclastic methanogenesis during thermophilic digestion of biowaste
Source: Microbiome. 2020 Jul 3;8:105. doi: 10.1186/s40168-020-00862-5 (PMC7334858; doi:10.1186/s40168-020-00862-5)
Supplement: Supplementary file 3 — Additional file 2. Supplementary Methods. [file 40168_2020_862_MOESM2_ESM.pdf]

Additional file 2 - Supplementary Methods

Syntrophic acetate oxidation replaces acetoclastic methanogenesis during thermophilic digestion of biowaste

Stefan Dyksma*, Lukas Jansen and Claudia Gallert

Faculty of Technology, Microbiology – Biotechnology, University of Applied Sciences Emden/Leer, Emden, Germany

* To whom correspondence should be addressed: Faculty of Technology, Microbiology – Biotechnology, University of Applied Sciences Emden/Leer, Emden, Germany

Tel +49 4921 807 1483, stefan.dyksma@hs-emden-leer.de

The metagenome analysis pipeline is separated into three separate but interlinked projects: KnuttReads2Bins, KnuttBinAnnotation and KnuttBinPhylo. KnuttReads2Bins performs read processing, DIAMOND [1] based annotation, read classification, assembly, contig classification and binning. We recommend to manually inspect the assembly/bins and make manual improvements. KnuttBinAnnotation takes bins as its input and applies the described set of annotation tools to them. KnuttBinPhylo can provide reference proteomes for supplied bins by using phylogenetic markers found in UniProt [2] proteomes. Every pipeline contains data preparation/extraction steps which produce easy parseable data files from every step. All data outputs are documented online, and every dependency and reference database are automatically downloaded and installed. The Snakmake [3] implementation allows users with Snakemake experience to easily adopt the pipelines to the specific needs. Further documentation can be found under **Knutt.org**.

KnuttReads2Bins

The first pipeline generates bins and read annotation data to allow for manual bin improvement and whole sample analysis. The additional dataset includes read and contig classification using SSU reads and coding gene reads.

Read preparation

The binning pipeline takes paired read FASTQ file as its input. For these files and following FASTQ files FASTQC [4] reports were generated. Additionally, an R script using the ShortRead [5] and Biostrings [6] library was used to construct the data for custom FASTQ data plots with ggplot [7] and plotly [8]. This included the quantiles and average of the read lengths in the file, the total base pair count, different kernel density estimations and a PHRED [9] score occurrence matrix giving the abundances of the score values in the cycles (positions) of the sequences. The density values were reported for the read GC contents, the read lengths and the mean read PHRED score. Kernel density estimation was performed with the R default parameters and was not trimmed.

Adapter trimming was performed with cutadapt [10]. The specified adapter was the Nextera 3' adapter sequence as a non-internal 3' adapter on both the R1 (-a) and R2 (-A) read files. The default parameters were used with the exception of the minimum overlap (-m), which was set to 6. Reads shorter than 20bp (--minimum-length) were discarded. Data on the cutadapt operation is read from its tab separated minimal report file (--report=minimal).

Merging was performed with BBmerge [11] using default parameters. Quality trimming on both ends after unsuccessful merging was enabled with a quality trim value of 10. Both the raw reads and the trimmed reads were merged. This was done to check the adapter output of BBmerge to

see, whether adapter trimming was successful and how it impacted merging performance. While BBmerge insertion detail file was used for data analysis, it lacks information on the ambiguous joins, therefore these statistics and others were read with regular expressions using R with the stringR [12] library from the BBmerge log file.

The reads for annotation were further processed. BBmask [13] replaced low complexity sections with a minimum entropy of 0.75 of pentamers in an 80bp window in the unmergeable R1 and merged reads with undetermined base calls. Cutadapt then trimmed the reads with quality trimming at cutoff 20 and undetermined base call trimming at the ends. The minimum length for the annotation reads was set at 100 bp, shorter ones were not used. For the classification FASTQ file the quality and low complexity trimmed merged and unmerged R1 were combined into a single query file. Data on the quality trimming process is also read from the cutadapt minimal report file.

Read Classification

Reads were classified with three different methods to provide abundance and diversity data. The small subunit rRNA (SSU) non-redundant database version 132 from the SILVA [14] project was used as reference database for BBmap [15]. Reads were mapped using default options and a minimum identity (minid) of 76%. The pre-aligned ARB file from the SILVA database was used as reference data for classification using SINA [16]. The result BAM file from BBmap was parsed with the Rsamtools R library and merged with the SINA results. For coding gene classification with Kaiju [17] we used the NCBI non redundant (nr) RefSeq. Kaiju was run with the default “greedy” mode with default parameters. The available taxonomy is based on the NCBI taxonomy. Sourmash [18] was used to calculate signatures for each sample. The k-mer size for this calculation is set to 31. The Genome Taxonomy Database (GTDB) taxonomy reference database 89 [19] is used during LCA classification. The results from all three tools are converted into a format compatible with Krona [20].

Read Annotation

Reads were further used as an input for DIAMOND [1] BLASTX against different protein databases. To minimize false positive results, the e-value filter was set to 10^{-5} and only the best hit with the lowest e-value for every query read was kept during result processing. A custom database was constructed to find formate dehydrogenase enzymes. This has been accomplished using a UniProtKB [2] query that searches for proteins with the relevant enzyme classes and KEGG [21,22] orthology terms. Another database was based on the hydrogenase enzymes from the HydDB [23] project. Carbohydrate active enzymes were identified using the latest CAZyDB [24] database version (07-31-2019) of the dbCAN [25] project.

Assembly

The MEGAHIT [26] assembler was used with the trimmed and merged reads, separately passing the unmerged reads as paired and the merged reads as single reads. To optimize for low coverage, high diversity samples, the k-mer increase between iterations was set to 10, starting at 27 and ending at 127. Otherwise default parameters were used, including the minimum k-mer multiplicity of 2 (--min-count) and the minimum output contig length of 200 (--min-contig-len).

To calculate the coverage depth for binning, the trimmed reads were mapped to the assembly using BBmap using similar parameters recommended for binning with METABAT2 [27]. Assembly quality statistics were calculated using metaQUAST [28] in Prokarya mode. The minimum contig length (-m) was set to 200. rRNA finding with Barnap (--rna-finding) and single copy ortholog search, similar to CheckM [29] with BUSCO [30] (-b) was enabled. Contig classification was performed with the CAT/BAT [31] workflow and sourmash. Sourmash is also applied to calculate the signatures of every contig (K set to 31). The same database for sourmash read classification was used for contig and bin classification.

Binning

Binning was performed with METABAT2 [27] using default parameters. The pseudo-random seed was fixed to 42. The completion and contamination data were calculated with the lineage specific workflow in CheckM [29]. CheckM was also used to calculate the tetra-nucleotide frequencies, depth data and composition data. The CAT Prodigal and DIAMOND results were reformatted to use them for the multi bin BAT workflow to taxonomically classify the bins. The fraction threshold below 50% allows for multiple taxonomic assignments for the same bin. Each bin was also classified again using sourmash.

Reporting

Most data processing tasks were performed with the data.table [32] R library or simple Python scripts. The final data aggregation steps in the workflow combined the data files from the different samples into a single file. The reports were based on RMarkdown [33] files and additionally use the plotly [8] and FlexDashboard [34] libraries for interactive single file HTML outputs. All reports contain file information like size, modification date and MD5 hash sum on all input files to minimize the risk of mixing different results from different runs and to indicate changes. Details on individual reports is given on www.knutt.org.

KnuttAnnotation

The second pipeline combines the annotation tools which were performed on the reconstructed bins. It combined the output files from every tool into gene format files (GFFs) for the contigs, ORFs and different tabular summary files.

MetaErg

MetaErg [35] was used for annotation of the contigs. It was executed with the 132 version of the SILVA database for SSU and LSU rRNA classification. The default minimum contig length was 200 base pairs and minimum ORF length was 180 amino acids. The Prodigal output from MetaErg was used for further ORF annotation tools. The output ORF FASTA file was split into multiple chunks for easier parallelization.

dbCAN

The dbCAN annotation steps were reimplemented with Snakemake and R for workflow integration and stability. The dbCAN version was similar to the version used for read annotation. A DIAMOND search was also performed against the transporter class database with an e-value of 10^{-10}. Hotpep [36] annotation was executed with the minimum number of unique k-mers set to 6 and the sum of the conserved k-mer frequencies to 2.6. The tool hmmscan [37] was executed with the CAZy models, the transcription factor models tf1/tf2 and a set of signal transduction protein models. E-value and coverage parameter are listed in Table S5. The search for carbohydrate active enzyme gene clusters (CGCs) was also reimplemented. The maximum number of spacer genes not having any dbCAN within a cluster was set to 1.

Table S5. E-value and coverage parameter

Model file	Description	E-value	Coverage
stp	dbCAN CAZy models	10^{-15}	35%
tf1/tf2	Transcription factors	10^{-4}	35%
stp	Signal transductors	10^{-4}	30%

HydDB

The HydDB online tool doesn’t provide an official API. Therefore, the HTTP calls between the data entry forms were emulated. This was done in a Python script. HTTP communication was

performed with the requests [38] package. The returned HTML pages were parsed with the BeautifulSoup [39] package. This was necessary to read the Cross-Site-Request-Forgery (CSRF) token and detect the fieldnames for resubmitting the downstream sequence of FeFe-hydrogenases for example. Data processing was done with the Pandas [40] python package and FASTA parsing with BioPython [41]. Candidates to be submitted to the HydDB classifier were selected with a RPS-BLAST search. An e-value of 10^{-10} was used for the search. For every query the longest, non-overlapping annotations were filtered. The filtered set of sequences was chunked and then submitted to the classifier.

KofamKOALA, InterProScan and eggNOG-mapper

For KEGG orthology annotation KofamKOALA [42] was used with default parameters on the ORFs. Module mapping and reconstruction was performed with a custom R script using the KEGGREST [43] library. Only modules with at least three blocks were allowed to have one block missing. The InterProScan [44] software version 5.40-77.0 with the Panther database version 14.1 was used with the Gene Ontology (GO), pathway and InterPro entry lookup. The eggNOG mapper was used with the database version 5.0.0 based on DIAMOND BLASTP searches. The seed ortholog search e-value threshold was set to 10^{-3} and the bit score threshold to 60. All ortholog types were used for annotation and the automatic scope detection.

KnuttBinPhylo

The KnuttBinPhylo pipeline produces a phylogenetic tree based on a concatenated alignment of different marker proteins, extracted from the bins and reference proteins in UniProt Proteomes [2]. The marker candidates are configurable and given as Pfam [45] family entries. The default selection is based on the category A set of markers compiled in the PhyloM bacteria set [46].

The hidden Markov models were downloaded directly from the Pfam database. Proteins with those markers annotated were queried from UniProtKB using a search that only allows for proteins included in non-redundant, non-excluded proteomes belonging to the *Archaea* or *Bacteria* superkingdom. Each set of reference marker proteins was then aligned using MAFFT [47–49] with the automatic algorithm (--auto) selection and support for unusual characters (--anysymbol). The markers were filtered, so that the selected markers cover at least 75% of the *Archaea*, *Bacteria* and “Unclassified” proteomes respectively. Proteomes were also filtered, requiring 50% of the selected markers. To resolve multiple marker occurrences in one proteome, the Jukes-Cantor [50] (JC) distances to a subsample of 1000 entries in the marker reference alignment were determined. If the fractional identity between two compared sequences was below or equal to the maximum for JC (95%), the distance was assumed to be infinite. Positions being gaps on both

sequences were ignored. The distances to the 1000 sample entries were summarized by calculating the median. This median was then used to sort the conflicting entries for each proteome and the lowest scoring entry was finally selected.

To analyze the bins, protein encoding genes were predicted with Prodigal [51] in anonymous/meta mode. HMMERs hmmsearch [37] with the gathering cutoff enabled searches for marker candidates in those metaproteomes. The hit regions were extracted. The marker hits in the bins were added to the respective marker reference alignment with MAFFTs add/keeplength option and FFT-NS-1 method. Bins were also required to have 50% of the selected markers and the same multiple hit resolution method was applied to the them. Missing marker sequences are filled with gap symbols while concatenating the marker sequences for the alignment sequence.

The tree was built with close and distant references. Selection of these references started by using the concatenated alignment to calculate the JC distance between the bins and all selected reference proteomes. The close references were the five closest references to the bin. Distant reference selection first took the 500 closest references and then calculated the JC distance between all those entries to construct a distance matrix. This distance matrix was used to perform hierarchical clustering with the R hclust “ward.D2” method and the resulting dendrogram was cut into 15 groups. From each of these groups the closest reference to the bin was used as a distant reference. For RAxML [52] a partitioning file was generated, separating the concatenated sequences. Each region was marked to be modeled with the LG model. RAxML tree calculations were performed with the “classic” RAxML using 100 rapid bootstraps for confidence on the best scoring maximum likelihood tree.

References

1. Buchfink B, Xie C, Huson DH. Fast and sensitive protein alignment using DIAMOND. Nat Methods. Nature Publishing Group; 2015;12:59–60.

2. The UniProt Consortium. UniProt: a worldwide hub of protein knowledge The UniProt Consortium. Nucleic Acids Res. 2019;

3. Köster J, Rahmann S. Snakemake-a scalable bioinformatics workflow engine. Bioinformatics. 2012;28:2520–2.

4. Andrews S. FastQC A Quality Control tool for High Throughput Sequence Data. 2018. Available from: http://www.bioinformatics.babraham.ac.uk/projects/fastqc/

5. Morgan M, Anders S, Lawrence M, Aboyoun P, Pagès H, Gentleman R. ShortRead: A bioconductor package for input, quality assessment and exploration of high-throughput sequence data. Bioinformatics. 2009

6. Pagès H, Aboyoun P, Gentleman R, DebRoy S. Biostrings: Efficient manipulation of biological strings. R Package Version 2460. 2017.

7. Wickham H. ggplot2: Elegant Graphics for Data Analysis. New York: Springer-Verlag New York; 2016.

8. Sievert C. plotly for R. 2018.

9. Ewing B, Green P. Base-calling of automated sequencer traces using phred. II. Error probabilities. Genome Res. 1998

10. Martin M. Cutadapt removes adapter sequences from high-throughput sequencing reads. EMBnet.journal. 2011

11. Bushnell B, Rood J, Singer E. BBMerge – Accurate paired shotgun read merging via overlap. PLoS ONE. 2017

12. Wickham H. Stringr: Modern, consistent string processing. R J. 2010

13. Bushnell B. BBmap. 2020. Available from: sourceforge.net/projects/bbmap/

14. Quast C, Pruesse E, Yilmaz P, Gerken J, Schweer T, Yarza P, et al. The SILVA ribosomal RNA gene database project: improved data processing and web-based tools. Nucleic Acids Res. Oxford University Press; 2013;41:D590-6.

15. Bushnell B. BBMap: a fast, accurate, splice-aware aligner. Jt. Genome Institute Dep. Energy. 2014.

16. Pruesse E, Peplies J, Glöckner FO. SINA: Accurate high-throughput multiple sequence alignment of ribosomal RNA genes. Bioinformatics. Narnia; 2012;28:1823–9.

17. Menzel P, Ng KL, Krogh A. Fast and sensitive taxonomic classification for metagenomics with Kaiju. Nat Commun. 2016;

18. Titus Brown C, Irber L. sourmash: a library for MinHash sketching of DNA. J Open Source Softw. 2016

19. Parks DH, Chuvochina M, Waite DW, Rinke C, Skarshewski A, Chaumeil P-A, et al. A proposal for a standardized bacterial taxonomy based on genome phylogeny. bioRxiv. 2018

20. Ondov BD, Bergman NH, Phillippy AM. Interactive metagenomic visualization in a Web browser. BMC Bioinformatics. 2011

21. Kanehisa M, Sato Y, Furumichi M, Morishima K, Tanabe M. New approach for understanding genome variations in KEGG. Nucleic Acids Res. 2019;

22. Kanehisa M. KEGG: Kyoto Encyclopedia of Genes and Genomes. Nucleic Acids Res. 2000

23. Søndergaard D, Pedersen CNS, Greening C. HydDB: A web tool for hydrogenase classification and analysis. Sci Rep 2016 6. 2016;6:34212.

24. Lombard V, Golaconda Ramulu H, Drula E, Coutinho PM, Henrissat B. The carbohydrate-active enzymes database (CAZy) in 2013. Nucleic Acids Res. 2014

25. Zhang H, Yohe T, Huang L, Entwistle S, Wu P, Yang Z, et al. DbCAN2: A meta server for automated carbohydrate-active enzyme annotation. Nucleic Acids Res. 2018

26. Li D, Luo R, Liu CM, Leung CM, Ting HF, Sadakane K, et al. MEGAHIT v1.0: A fast and scalable metagenome assembler driven by advanced methodologies and community practices. Methods. 2016.

27. Kang DD, Li F, Kirton E, Thomas A, Egan R, An H, et al. MetaBAT 2: An adaptive binning algorithm for robust and efficient genome reconstruction from metagenome assemblies. PeerJ. 2019

28. Mikheenko A, Saveliev V, Gurevich A. MetaQUAST: Evaluation of metagenome assemblies. Bioinformatics. 2016

29. Parks DH, Imelfort M, Skennerton CT, Hugenholtz P, Tyson GW. CheckM: assessing the quality of microbial genomes recovered from. Genome Res. 2015

30. Simão FA, Waterhouse RM, Ioannidis P, Kriventseva E V., Zdobnov EM. BUSCO: Assessing genome assembly and annotation completeness with single-copy orthologs. Bioinformatics. 2015

31. Von Meijenfeldt FAB, Arkhipova K, Cambuy DD, Coutinho FH, Dutilh BE. Robust taxonomic classification of uncharted microbial sequences and bins with CAT and BAT. Genome Biol. 2019

32. Dowle M, Srinivasan A. data.table: Extension of `data.frame`. 2019.

33. Xie Y, Allaire JJ, Grolemund G. R Markdown: The Definitive Guide. Boca Raton, Florida: Chapman and Hall/CRC; 2018.

34. Iannone R, Allaire JJ, Borges B. flexdashboard: R Markdown Format for Flexible Dashboards. 2018.

35. Dong X, Strous M. An Integrated Pipeline for Annotation and Visualization of Metagenomic Contigs. Front Genet. 2019

36. Busk PK, Pilgaard B, Lezyk MJ, Meyer AS, Lange L. Homology to peptide pattern for annotation of carbohydrate-active enzymes and prediction of function. BMC Bioinformatics. 2017

37. Finn RD, Clements J, Eddy SR. HMMER web server: Interactive sequence similarity searching. Nucleic Acids Res. 2011

38. Reitz K, Benfield C, Cordasco IS, Prewitt N. Requests. 2019.

39. Richardson L. Beautiful soup. Crummy Site. 2013

40. McKinney W. Data Structures for Statistical Computing in Python. Proc 9th Python Sci Conf. 2010

41. Cock PJA, Antao T, Chang JT, Chapman BA, Cox CJ, Dalke A, et al. Biopython: Freely available Python tools for computational molecular biology and bioinformatics. Bioinformatics. 2009

42. Aramaki T, Blanc-Mathieu R, Endo H, Ohkubo K, Kanehisa M, Goto S, et al. KofamKOALA: KEGG ortholog assignment based on profile HMM and adaptive score threshold. bioRxiv. 2019

43. Tenenbaum D. KEGGREST: Client-side REST access to KEGG. 2019.

44. Huerta-Cepas J, Forslund K, Coelho LP, Szklarczyk D, Jensen LJ, Von Mering C, et al. Fast genome-wide functional annotation through orthology assignment by eggNOG-mapper. Mol Biol Evol. 2017

45. El-Gebali S, Mistry J, Bateman A, Eddy SR, Luciani A, Potter SC, et al. The Pfam protein families database in 2019. Nucleic Acids Res. 2019

46. Institut Pasteur Groupe d'Inférence Phylogénétique. PhyloM: bacteria. giphy.pasteur.fr. 2016. Available from: http://giphy.pasteur.fr/PhyloM/bacteria/

47. Katoh K, Standley DM. MAFFT multiple sequence alignment software version 7: Improvements in performance and usability. Mol Biol Evol. 2013

48. Katoh K, Standley DM. MAFFT: Iterative refinement and additional methods. Methods Mol Biol. 2014

49. Katoh K, Frith MC. Adding unaligned sequences into an existing alignment using MAFFT and LAST. Bioinformatics. 2012

50. Jukes TH, Cantor CR. Evolution of protein molecules. Mamm. Protein Metab. III. New York: Academic Press; 1969.

51. Hyatt D, Chen GL, LoCascio PF, Land ML, Larimer FW, Hauser LJ. Prodigal: Prokaryotic gene recognition and translation initiation site identification. BMC Bioinformatics. 2010

52. Stamatakis A. RAxML version 8: A tool for phylogenetic analysis and post-analysis of large phylogenies. Bioinformatics. 2014
